# Hemodynamic Analysis Shows High Wall Shear Stress Is Associated with Intraoperatively Observed Thin Wall Regions of Intracranial Aneurysms

**DOI:** 10.3390/jcdd9120424

**Published:** 2022-11-29

**Authors:** Sricharan S. Veeturi, Tatsat R. Patel, Ammad A. Baig, Aichi Chien, Andre Monteiro, Muhammad Waqas, Kenneth V. Snyder, Adnan H. Siddiqui, Vincent M. Tutino

**Affiliations:** 1 Canon Stroke and Vascular Research Center, University at Buffalo, Buffalo, NY 14203, USA; 2 Department of Mechanical and Aerospace Engineering, University at Buffalo, Buffalo, NY 14260, USA; 3 Department of Neurosurgery, University at Buffalo, Buffalo, NY 14260, USA; 4 Department of Radiology, University of California Los Angeles, Los Angeles, CA 90095, USA; 5 Department of Pathology and Anatomical Sciences, University at Buffalo, Buffalo, NY 14260, USA; 6 Department of Biomedical Engineering, University at Buffalo, Buffalo, NY 14260, USA

**Keywords:** intracranial aneurysm, computational fluid dynamics (CFD), intraoperative video, wall shear stress (WSS), relative residence time (RRT), aneurysm wall characterization

## Abstract

Background: Studying the relationship between hemodynamics and local intracranial aneurysm (IA) pathobiology can help us understand the natural history of IA. We characterized the relationship between the IA wall appearance, using intraoperative imaging, and the hemodynamics from CFD simulations. Methods: Three-dimensional geometries of 15 IAs were constructed and used for CFD. Two-dimensional intraoperative images were subjected to wall classification using a machine learning approach, after which the wall type was mapped onto the 3D surface. IA wall regions included thick (white), normal (purple-crimson), and thin/translucent (red) regions. IA-wide and local statistical analyses were performed to assess the relationship between hemodynamics and wall type. Results: Thin regions of the IA sac had significantly higher WSS, Normalized WSS, WSS Divergence and Transverse WSS, compared to both normal and thick regions. Thicker regions tended to co-locate with significantly higher RRT than thin regions. These trends were observed on a local scale as well. Regression analysis showed a significant positive correlation between WSS and thin regions and a significant negative correlation between WSSD and thick regions. Conclusion: Hemodynamic simulation results were associated with the intraoperatively observed IA wall type. We consistently found that elevated WSS and WSS_Norm_ were associated with thin regions of the IA wall rather than thick and normal regions.

## 1. Introduction

Intracranial Aneurysms (IAs) are focal dilations on the cerebral vasculature that can eventually grow and rupture, causing devastating hemorrhagic strokes [1,2]. Human and animal studies have demonstrated that cerebral hemodynamics plays a key role in the natural history of IA formation, growth, and rupture [3,4]. Nascent IAs preferentially form in regions of the Circle of Willis that experience aberrant hemodynamics, namely areas of high wall shear stress (WSS), the frictional force exerted by blood on the vessel wall [5]. As these lesions grow, the intra-aneurysmal hemodynamics change and propagate different pathobiological processes or cascades in the wall. It has been theorized that high WSS (e.g., from impinging flow) leads to mural cell-mediated destructive remodeling and wall degeneration, while low WSS (e.g., in low-flow regions) leads to inflammatory cell-mediated remodeling and plaque buildup [6]. However, biological investigation of these processes in humans is not possible, and there are no true animal models of IA growth and rupture to test this. 

In order to evaluate the relationship between intra-aneurysmal hemodynamics and local wall pathobiology, studies have analyzed intraoperative imaging taken during IA clipping, and compared the observed wall appearance with IA flow generated from computational fluid dynamics (CFD) performed on segmented angiography [7,8]. Several studies have found that thin aneurysmal walls, with a red and sometimes translucent appearance, are associated with high-velocity blood flow and elevated WSS. This relationship has been further substantiated by animal models in which high flow (namely high WSS and a positive WSS gradient) has been induced to generate wall degeneration and aneurysmal remodeling at bifurcations in the Circle of Willis in rabbits, rats, and mice [9]. On the other hand, aneurysmal regions appearing to be thick and atherosclerotic, with a white/tan or yellow color, have been reported to be associated with a slower flow and low WSS [10,11,12]. Slow, recirculating flow has been shown to promote the formation of atherosclerotic plaques, which are generally associated with the infiltration of immune cells and pathological vascular remodeling, hallmarks of IA natural history [6,13]. 

Over the last 10 years, however, several other reports have generated conflicting findings [14,15]. One potential reason for these contradictions is the wide variations in intraoperative image analysis techniques [16]. Most studies have used qualitative approaches to delineate different wall regions (e.g., manual marking), while others have used rudimentary image processing methods (such as pixel thresholding). This could lead to high inter-operator variation and lower image-to-image agreement, as well as precluding local analysis of the flow–wall relationship. 

In this study, we hypothesized that different local hemodynamic environments in the IA sac could elicit different biological responses by the IA wall and, consequently, different wall presentations, as observed on intra-operative images. The goal of the current study is to identify different wall types objectively, as manifested in intraoperative images and investigate the underlying relationship with the local hemodynamics of the IA wall. To this end, we identified different wall types objectively using a machine-learning-based approach and then performed CFD on these cases to quantify local hemodynamics throughout the aneurysm sac. We then evaluated the potential correlation between local hemodynamics and different wall appearance. Statistical analysis was performed, investigating both the global (entire IA sac) and local association. 

## 2. Methods

### 2.1. Patient Population

This retrospective study was approved by the institutional review board at the University at Buffalo (STUDY00006058). Patient consent was waived. We retrospectively collected images and intraoperative videos of 28 IAs from 27 adult patients who underwent open-skull surgery at the Gates Vascular Institute in Buffalo, NY, between January 2010 and March 2016. Upon review of intraoperative videos and angiographic imaging, cases were removed from the following analyses if a clear image of the majority of the IA was not present or if the medical imaging could not be completely segmented. Examples of such cases are shown in Supplemental Figure S1.

### 2.2. Delineation of Different IA Wall Types

**Figure 1:**
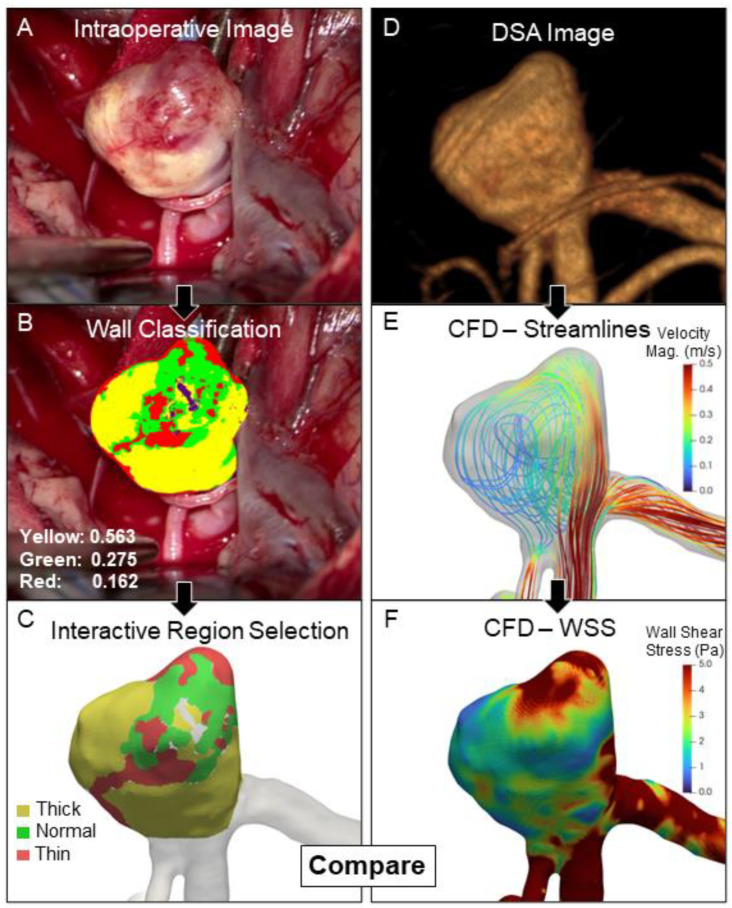
The overall workflow for exploring the relationship between hemodynamics and intraoperatively observed wall type: A screenshot of the best view of the aneurysm from the video is obtained for further analysis (**A**). For the classification of different wall types, we used a machine-learning-based tool trained by a clinician. This gives a percentage of different areas marked by the clinician, as shown in the wall classification image (**B**). The different regions are then mapped onto the 3D surface (**C**). The DSA image is segmented and used for CFD analysis (**D**,**E**). The hemodynamic variables from the mapped regions are then exported for further statistical and regression analysis (**F**). Abbreviations: CFD = computational fluid dynamics, DSA = digital subtraction angiography, Mag. = magnitude, WSS = wall shear stress.

To identify different regions of the IA wall objectively with intraoperative imaging, we used Orbit image analysis (www.orbit.bio) [17]. This open-source image processing software uses a machine learning algorithm (support vector machines) to recursively train a classifier for *n* number of different regions based on training data of example regions defined by user input. With this software, a trained neurosurgeon manually marked the training data for four distinct IA wall regions that had a different appearance on intraoperative imaging. These included: thick/atherosclerotic regions (tan/yellowish), normal vessel regions (purple-crimson), thin/translucent regions (dark red), and regions of imaging artifact due to surgical lighting (bright white reflections). Once the classifier was trained with data from all images, we used it to classify the IA wall appearance in all cases, as exemplified in Figure 1B. This pipeline also output details on the proportion of pixels in each category. We used an open-source platform (ParaView) to overlay the classification image and interactively orient the 3D surface model of the reconstructed IA. We then selected the regions of interest on the IA sac in the different marked regions in the background classification image resulting in a surface file with different faces, as shown in Figure 1C. Care was taken to exclude the pixels with reflection artifacts, as shown in purple in Figure 1B. We normalized the proportions of the remaining classes so the sum of the other three classes would be unity in each case and reported the percentage of each region for every IA.

### 2.3. Patient-Specific Hemodynamics

To analyze intra-aneurysmal blood flow, we performed CFD using the open source platform OpenFOAM v.6, as described previously [18]. Briefly, DSA images were segmented using the VMTK (www.vmtk.org) open-source software. For IAs located in the anterior Circle of Willis, images were segmented beginning at the cavernous segment of the ICA, and for IAs located in the posterior Circle of Willis, images were segmented beginning at the vertebral arteries. The segmented images were then prepared for CFD analysis using Meshmixer. CFMesh was used to generate a polyhedral mesh for the geometries with a base size of 0.2 mm and 4 prism layers with 0.1mm thickness for the first layer and a 1.2 thickness ratio for successive layers. This resulted in an average mesh of 2.8 ± 0.7 million polyhedral elements across all cases.

For the CFD, we assumed blood to be a Newtonian fluid with a viscosity of 0.0035 Ns/m^2^ and a density of 1060 kg/m^3^ and the vessels to be rigid. The inlet boundary conditions included a constant cycle averaged velocity assumption of 24 cm/s [19] at the internal carotid artery (ICA). This figure was dampened by 30% for aneurysms located at the middle cerebral artery (MCA) of the anterior communicating artery (AComm) to account for the transit from the cervical to the cavernous segments, as described elsewhere [20]. For the outlet boundary conditions, we computed the flow split, based on the diameters at the bifurcations of arteries following Chnafa et al. [21]. We assumed a time step of 0.005 seconds and ran the simulation for 2 cardiac cycles. Flow variables recorded every 0.01 seconds were exported, and the 2nd cardiac cycle was used for analysis. For our analysis, we computed the time-averaged wall shear stress on the wall of the aneurysm (WSS), normalized WSS (WSS_Norm_), oscillatory shear index (OSI), relative residence time (RRT), wall shear stress divergence (WSSD) and transverse wall shear stress (TransWSS), the definitions of which are provided in the online supplement.

### 2.4. Image Co-Registration

For co-registration, the 2D intraoperative image that displayed the largest proportion of the aneurysm and the 3D CFD domain were imported into the open-source image processing software ParaView. Using the 2D intraoperative image as a background, the 3D IA was manipulated until it matched the orientation of the background IA under the supervision of an experienced neurosurgeon. In brief, first, the 3D geometry was rotated so that visible landmarks, e.g., parent vessel or daughter branches, were best aligned with the background image. The geometry was then rotated along that axis such that the projection of the 3D geometry matched the contour of the background IA. For co-mapping, the regions identified by Orbit image analysis were then projected onto the oriented 3D geometry, as shown in Figure 1E. The hemodynamic values at each of the classified mesh nodes across all regions were exported for further analysis. 

### 2.5. Statistical Analysis

Statistical analyses were performed to assess the differences in wall hemodynamics across each different type of IA region. The Shapiro–Wilk test was performed to determine if the data were normally distributed or not. For normally distributed data, ANOVA was performed to assess multiclass differences in variation, while the Kruskal–Wallis test was used for non-normally distributed data. For pairwise univariate comparisons (i.e., for thick vs. thin regions, thick vs. normal regions, and normal vs. thin regions), A Student’s *t*-test was used for normally distributed data, while a Mann–Whitney U test was used for non-normally distributed data. Multiclass and univariate analyses were performed globally, i.e., on the median value of hemodynamic parameters for each region in every IA, and locally, at all of the image nodes in different regions of each aneurysm. A *p*-value of <0.05 was considered significant. Due to the large observation size of the local analysis, we also performed Cohen’s D test to quantify the effect size. A D > 0.4 was considered to be notable. 

To explore the global relationship between IA hemodynamics and wall appearance, we performed Pearson correlation analysis. In cases with >30% of their sac area visible on intraoperative imaging, we recorded the median hemodynamic parameters and percentage of the sac covered by each different wall “type” from Orbit image analysis. We quantified the degree of correlation using Pearson’s Correlation Coefficient (PCC) and the Wald test (a correlation with a p-value<0.05 was considered significant). A 1 ≥ |PCC| ≥ 0.8 represented a ‘very strong’ correlation, 0.79 ≥ |PCC| ≥ 0.6 represented a ‘strong’ correlation, 0.59 ≥ |PCC| ≥ 0.4 represented a ‘moderate’ correlation, 0.39 ≥ |PCC| ≥ 0.2 represented a ‘weak’ correlation, and |PCC| < 0.19 represented a ‘very weak’ or no correlation [22].

## 3. Results

Images of a total of 28 IAs from 27 patients were collected for this study. However, 13 aneurysms were removed from the analysis because a majority of their IA could not be seen in the intraoperative video or there was poor segmentation from the DSA. As outlined in the flow chart in Supplemental Figure S2, a total of 15 aneurysms from 14 patients were included in our final analysis. Of these, six were located at the ICA, three were located at the AComm, and six were located at the MCA. 

### 3.1. Higher WSS in Thin and High RRT in Thick IA Wall Regions

**Figure 2:**
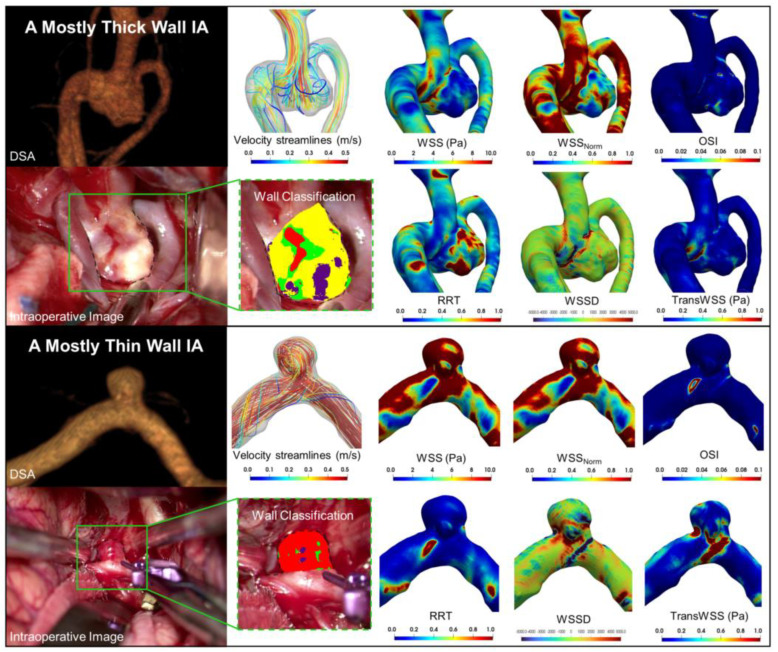
Visualization of cases with different wall types: Representative cases of IAs with mostly thick-walled as well as thin-walled regions, along with contours of their respective hemodynamic variables. The DSA images are displayed on the top left, and the intraoperative classification of the images are displayed on the bottom left. Thin-walled IAs had high WSS, WSS_Norm_, WSSD and TransWSS, and thick-walled IAs had a high RRT. Abbreviations: IA = intracranial aneurysm, DSA = digital subtraction angiography, WSS = wall shear stress, WSSNorm = normalized wall shear stress, OSI = oscillatory shear index, RRT = relative residence time, WSSD = wall shear stress divergence, TransWSS = transverse wall shear stress.

**Figure 3:**
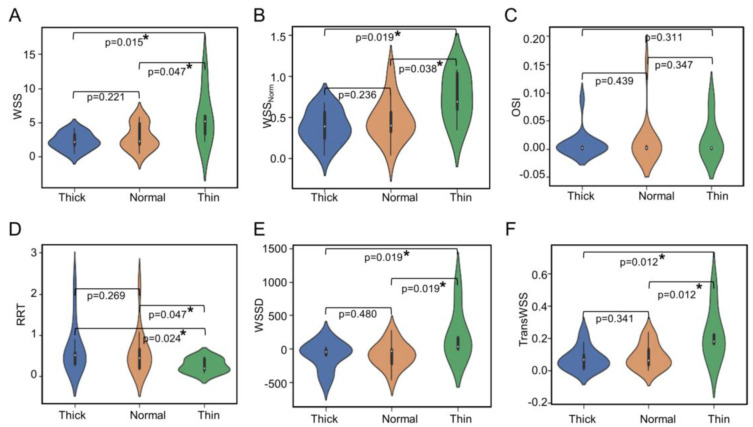
Pairwise univariate analysis of hemodynamic variables between different wall types across all IA sacs: The violin plots of median values of WSS (**A**), WSSNorm (**B**), OSI (**C**), RRT (**D**), WSSD (**E**), and TransWSS (**F**) across all IA sacs. All the significant differences (*p* < 0.05) are marked with an asterisk (*). Abbreviations: WSS = wall shear stress, WSSNorm = normalized wall shear stress, OSI = oscillatory shear index, RRT = relative residence time, WSSD = wall shear stress divergence, TransWSS = transverse wall shear stress.

Two representative cases, one with predominantly thick (white) regions, and another case with predominantly thin (red) regions, along with their hemodynamic characteristics, are shown in Figure 2. Qualitatively, we observe that the case with thick regions had lower WSS, WSS_Norm_, and OSI and a higher RRT. The median values and interquartile ranges (IQR) of the median hemodynamic variables in each type of wall region across all IAs are presented in Table 1. Based on our multiclass univariate analysis, we found that WSS_Norm_ and TransWSS were significantly different in at least one of the IA wall types across all samples (*p* = 0.029 and 0.049, respectively). The violin plots in Figure 3 show the distribution of the median values of hemodynamic metrics for different wall types. Along with the median and IQR, the violin plot gives information on the distribution and density of the data. As in multiclass comparison, the pairwise univariate analysis also showed that both median WSS_Norm_ and TransWSS were statistically significantly higher in thin IA wall regions than in thick and normal regions. Additionally, this analysis also revealed that median WSS and WSSD were significantly higher in regions of thin IA walls vs. thick and normal regions. Conversely, the RRT was significantly lower in the thin areas compared to the thick and normal areas. There was no significant difference in OSI among the different regions. Furthermore, none of the hemodynamic metrics were significantly different between the thick and normal regions of the IA sacs.

We also performed these statistical analyses at a local level, i.e., on a node-to-node basis. Here, we analyzed many data points; ~127,000 points from the thick regions, ~25,000 from the thin regions, and ~93,000 from the normal regions. As shown in Supplemental Table S1, multiclass and pairwise univariate analyses showed significant differences in all hemodynamic parameters between the regions (all *p* < 0.05). However, as these results may be skewed by the large sample size, we further calculated Cohen’s D for the pairwise analysis (Supplemental Figure S3). Similar to the comparisons of medians, this analysis showed higher WSS, higher WSS_Norm_, and lower RRT in thin regions, with a sufficient effect size (D > 0.40) compared to both thick and normal regions. TransWSS was also higher in thin regions with good effect size (D = 0.415), but only in comparison to normal regions. 

### 3.2. Correlation between Hemodynamic Variables and Percentage Wall Type

To investigate the relationship further between median IA sac hemodynamic values and the amount of IA wall comprised of either thin or thick wall types, we performed a Pearson correlation analysis on cases where a substantial proportion of the wall was visible. As shown in Supplemental Figure S4, we found that the percentage area of thin regions had a significant (*p* = 0.010) and very strong positive correlation (PCC = 0.959) with WSS. There was also a strong negative correlation (PCC = −0.735) between the percentage area of thin regions and RRT, albeit the trend was not statistically significant, *p* = 0.157. On the other hand, the percentage area of thick regions had a significant (*p* = 0.041) and strong negative correlation (PCC = −0.728) with WSSD (see Supplemental Figure S5). All other correlations were not strong or significant by the Wald’s test.

## 4. Discussion

Studying the relationship between blood flow forces and local aneurysm pathobiology can help us understand the complex process of IA natural history. In this study, we explored the local and global relationship between different IA wall types observed on intraoperative imaging and aneurysmal hemodynamics from 3D image-based CFD. We found that thin, red regions of the aneurysm sac were exposed to significantly higher WSS, WSS_Norm_, WSSD, and TransWSS than normal and thick atherosclerotic regions. Conversely, the thicker, white regions in the IA sac tended to co-locate with significantly higher RRT than in the thin regions. In general, when comparing node-to-node between mapped wall type and 3D CFD, these trends were also observed on a local scale. Regression analysis of median hemodynamic variables and percentage area of thin and thick regions showed a further significant positive correlation between WSS and thin regions and a significant negative correlation between WSSD and thick regions.

Over the last 10 years, the approach of comparing image-based CFD and intraoperative imaging has been used by several IA research groups. In a comprehensive literature review, we identified 17 such studies that reported findings between 2013 and 2022 (see Supplemental Table S2). Oftentimes, these studies, like ours, analyzed smaller datasets of IAs, ranging from n = 4 to n = 65 (mean sample size of 25). They also tended to focus on the analysis of WSS or its derivatives (15 studies). Similar to our findings, three of these studies found significantly higher WSS in thin regions of the IA wall [7,10,23]. Other reports (5 studies) also echoed this trend [11,12,24]. For example, they showed that greater RRT and lower WSS were related to thicker walls or that WSSD was associated with thinner walls. However, these studies did not perform quantitative analyses to demonstrate statistical significance. 

Contradictory results have also been reported among these 17 studies. Specifically, three of them report significantly lower WSS at thin regions of the IA sac [8,14,25]. These conflicting findings may be the result of small sample sizes (three of the seventeen studies analyzed sample sizes under n = 10) or inconsistent CFD results due to variability in assumptions or boundary conditions. These factors aside, we believe a major limitation of the previous studies is the lack of objective quantification methods to identify thin or thick regions accurately on intraoperative images. The majority of previous studies (11) used manual marking of regions identified by a single user, while others used simple RGB thresholding on the images to identify regions (6). This could lead to biased results and inaccurate identification of regions (especially around boundaries) and does not account for image artifact in a standardized fashion. 

In the current study, we used an objective image analysis pipeline for the standardized identification of different vascular thicknesses and image artifacts, which enabled the more accurate and objective transfer of thin and thick regions to the 3D geometry and association with CFD. Based on multiple statistical analyses (multiclass and pairwise analyses and Pearson correlation), we consistently found that elevated WSS and WSS_Norm_ were associated with thin, red regions of the IA wall. Animal and human studies have demonstrated that high WSS plays a key role in IA initiation and may persist in the aneurysm sac during aneurysm growth and development. Physiologically, abnormally elevated WSS has been shown to propagate endothelial damage and turnover. When the local WSS exceeds certain thresholds, endothelial mechanotransduction initiates mural cell production of matrix metalloproteinases. This leads to massive internal elastic lamina damage and apoptosis, which result in medial layer degeneration and thinning. Thus, it makes sense that higher WSS is associated with regions of the IA that are extremely degraded, acellular, and thin. We also found higher levels of other WSS-related parameters, namely WSSD and TransWSS, and lower levels of RRT in the thin red regions. Such association further indicates that degenerated, thinned regions of the aneurysm wall are related to fast, potentially impinging, and circulatory flow, which locally stretches the luminal surface. While little is known about the response of vascular cells under TransWSS and DWSS, greater internal stretching can induce stress responses, endothelial turnover, and vascular remodeling responses, including extracellular matrix degradation, which, when gone awry, may contribute to wall thinning in these regions. 

On the other hand, this analysis demonstrated that the white, atherosclerotic-appearing regions had a lower WSS (including WSS_Norm_, WSSD, and TransWSS) and a higher RRT. Studies have demonstrated that abnormally low WSS flow can promote endothelial cell inflammation and increase the production of reactive oxygen species. This can increase the recruitment of chemotaxis of inflammatory cells and increase luminal permeability. Once infiltrated into the IA wall, inflammatory cells, primarily macrophages and T cells, have been shown to produce massive amounts of proteases that can lead to matrix degeneration. Higher RRT in the white regions of the IA also signifies a higher blood residence time at that location. This could increase circulating immune cell trafficking to these regions, and thus lead to greater infiltration of inflammatory cells. We suspect these flow conditions and resultant processes mimic atherosclerotic plaque genesis, which can also include vascular smooth muscle infiltration into intimal hyperplasia and proliferation. Interestingly, there were no statistically significant differences in flow at the white regions vs. normal wall regions, unlike thin, red regions with a significantly different flow to both thick and normal areas. This suggests a much stronger, local relationship between high flow and thin, red regions, but not for thick, white regions. 

This study has several limitations. First, our sample size was relatively small, which could limit confidence in our findings. Future studies in larger sample sizes are warranted. Second, we assumed the arterial walls to be rigid and the blood to be a Newtonian fluid. We also used generic assumptions for CFD, since patient-specific flow information was not available. The use of patient-specific boundary conditions would make the CFD simulations more accurate. Third, while we used a rigorous approach for mapping different regions of the 2D intraoperative imaging to the 3D IA geometry, there may be inaccuracies in co-mapping wall type to CFD. The 3D volume is captured from the lumen, whereas the 2D image is a representation of the outer IA wall, which is not likely to be the same shape as the lumen, particularly for the thicker regions. Furthermore, the IA may have changed shape during surgery. Fourth, we used a machine learning strategy for the classification of wall appearance. While this method is semi-automated and objective, the models are still trained by a clinician, and therefore there may be subjectivity in the ground truth classes. Furthermore, it only classified wall structures into the four identified groups (thin, thick, normal, and artifact) without the ability to identify other types of wall appearances. Fifth, we assumed that the white/tan or yellow regions correspond to thicker regions of the IA that may be atherosclerotic or hyperplastic, and the red regions were thinned or degraded areas. Additional histologic studies are needed to demonstrate these claims empirically. Lastly, there are other hemodynamic parameters that could be explored. However, we limited our investigation to those previously shown to be significantly associated with IA natural history [5]. 

## 5. Conclusions

In this study, we found that aneurysmal hemodynamics were locally associated with an intraoperatively observed IA wall type. Specifically, we consistently observed that elevated WSS and WSS_Norm_ were associated with thin regions of the IA wall rather than thick and normal regions. Furthermore, RRT was also significantly higher in thick regions than the thin, red areas. Based on our literature review, these findings were in line with many others published in the current literature. Future studies in larger cohorts are needed to validate these findings. 

## Figures and Tables

**Table 1:** Median values and IQRs of different hemodynamics metrics in different wall regions across all aneurysm sacs *.

Parameter	Thick (Median ± IQR)	Normal (Median ± IQR)	Thin (Median ± IQR)	*p*-Value
WSS (Pa)	2.201 ± 1.507	2.268 ± 2.939	5.236 ± 2.625	0.074
WSS_Norm_	0.388 ± 0.317	0.400 ± 0.236	0.687 ± 0.435	0.029 *
OSI	0.002 ± 0.003	0.002 ± 0.003	0.002 ± 0.002	0.853
RRT (Pa^−1^)	0.517 ± 0.269	0.444 ± 0.454	0.191 ± 0.264	0.104
WSSD (Pa/m)	−41.158 ± 81.062	−35.280 ± 213.057	31.912 ± 165.988	0.069
TransWSS (Pa)	0.068 ± 0.077	0.064 ± 0.092	0.182 ± 0.086	0.049 *

* Median values of the median hemodynamic value at each wall type across all aneurysms. Abbreviations: WSS = wall shear stress, WSS_Norm_ = normalized wall shear stress, OSI = oscillatory shear index, RRT = relative residence time, WSSD = wall shear stress divergence, TransWSS = transverse wall shear stress, IQR = interquartile range.

## Data Availability

The data presented in this study are available on request from the corresponding author. The data are not publicly available due to continued analysis by the corresponding author’s research team and IRB restrictions.

## Supplementary Materials

The following supporting information can be downloaded at: https://www.mdpi.com/article/10.3390/jcdd9120424/s1, Table S1: Median values and inter-quartile ranges of different hemodynamics metrics in different wall regions on a local node-to node basis across all aneurysm sacs; Table S2: Detailed literature review of past studies exploring the relationship between hemodynamics and different IA wall types as observed intraoperatively; Figure S1: Examples of cases that were removed from analysis; Figure S2: Data availability flow chart; Figure S3: Pairwise univariate analysis of node-to-node hemodynamic variables between different wall types across all IA sacs; Figure S4: Regression analysis between hemodynamic variables and thin regions; Figure S5: Regression analysis between hemodynamic variables and thick regions.
