# Supplementary material for: Hemodynamic Analysis Shows High Wall Shear Stress Is Associated with Intraoperatively Observed Thin Wall Regions of Intracranial Aneurysms
Source: J Cardiovasc Dev Dis. 2022 Nov 29;9(12):424. doi: 10.3390/jcdd9120424 (PMC9780790; doi:10.3390/jcdd9120424)
Supplement: Supplementary file 1 [file jcdd-09-00424-s001.zip › jcdd-1992258-supplementary.pdf]

Supplemental Information for: Hemodynamic Analysis Shows High Wall Shear Stress is Associated with Intraoperatively-Observed Thin Wall Regions of Intracranial Aneurysms

Sricharan S. Veeturi MS[1,2], Tatsat R. Patel PhD[1,2], Ammad A. Baig MBBS[1,3], Aichi Chien PhD[6], Andre Monteiro MD[1,3], Muhammad Waqas MBBS[1,3], Kenneth V. Snyder MD PhD[1,3], Adnan H. Siddiqui MD PhD[1,3], *Vincent M. Tutino PhD[1,2,3,4,5]

Affiliations: [1]Canon Stroke and Vascular Research Center; [2]Department of Mechanical and Aerospace Engineering, [3]Department of Neurosurgery, [4]Department of Pathology and Anatomical Sciences, [5]Department of Biomedical Engineering, University at Buffalo, Buffalo, NY, USA

[6]Department of Radiology, University of California Los Angeles, Los Angeles, CA, USA

***Corresponding author**:

Vincent Tutino, PhD

Canon Stroke and Vascular Research Center

Clinical and Translational Research Center

875 Ellicott Street

Buffalo, NY 14214 USA

E-mail: vincentt@buffalo.edu

Phone: (716) 829-5400

Fax: (716) 854-1850

Supplemental Methods

Definitions of hemodynamic parameters

<u>*Time averaged wall shear stress (WSS):*</u> WSS is the average wall shear stress on the aneurysm sac. WSS_i is the instantaneous wall shear stress vector. T is the duration of the cardiac cycle.

$$WSS = \frac{1}{T}\left|\int_0^T WSS_i\right| dt$$

<u>*Normalized wall shear stress (WSS_{Norm}):*</u> WSS_{Norm} is the ratio of average wall shear stress on the aneurysm sac to the average wall shear stress on the parent artery. $WSS_{Aneurysm}$ is the average wall shear stress over the aneurysm sac and $WSS_{Parent\ Vessel}$ is the wall shear stress on the parent vessel upstream of the aneurysm.

$$WSS_{Norm} = \frac{WSS_{Aneurysm}}{WSS_{Parent\ Vessel}}$$

<u>*Oscillatory shear index (OSI):*</u> OSI quantifies the temporal variations of the wall shear stress vectors. Here $\left|\int_0^T WSS_i dt\right|$ represents the magnitude of the time averaged wall shear stress over the cardiac cycle and $\int_0^T |WSS_i| dt$ is the average of the magnitude of wall shear stress vectors over the cardiac cycle.

$$OSI = \frac{1}{2}\left\{1 - \frac{\left|\int_0^T WSS_i dt\right|}{\int_0^T |WSS_i| dt}\right\}$$

<u>*Relative residence time (RRT):*</u> RRT quantifies the near wall residence time of blood.

$$RRT = \frac{1}{\frac{1}{T}\left|\int_0^T WSS_i dt\right|} = \frac{1}{(1 - 2 \times OSI) \times WSS}$$

<u>*Wall Shear Stress Divergence (WSSD):*</u> WSSD quantifies the spatial variations in the time averaged wall shear stress vectors. Positive WSSD values signify stretching of the tissue and negative values indicate compression.

$$WSSD = \Delta.\tau = \frac{\partial \tau_x}{\partial x} + \frac{\partial \tau_y}{\partial y} + \frac{\partial \tau_z}{\partial z}$$

<u>*Transverse Wall Shear Stress (TransWSS):*</u> TransWSS quantifies the multi-direction force on the wall. Disturbed flow where the WSS vectors do not remain parallel to a single axis have non-zero WSS components in a direction normal to the temporal mean WSS vector.

$$TransWSS = \frac{1}{T}\int_0^T \vec{\tau}_i \left|\left(\vec{n} \times \frac{\vec{\tau}_{mean}}{|\vec{\tau}_{mean}|}\right)\right| dt$$

Where $\vec{n}$ represents the normal to the arterial surface and $\vec{\tau}_i$ is the instantaneous wall shear stress vector.

Supplemental Tables

Table S1: Median values and inter-quartile ranges (IQR) of different hemodynamics metrics in different wall regions on a local node-to node basis across all aneurysm sacs.*

Parameter	Thick (Median ± IQR)	Normal (Median ± IQR)	Thin (Median ± IQR)	*p*-value
WSS (Pa)	1.642 ± 2.265	1.651 ± 2.743	2.882 ± 3.335	<0.001 *
WSS_{Norm}	0.284 ± 0.455	0.284 ± 0.486	0.462 ± 0.528	<0.001 *
OSI	0.003 ± 0.022	0.004 ± 0.010	0.004 ± 0.037	<0.001 *
RRT (Pa^{-1})	0.694 ± 1.464	0.704 ± 1.561	0.381 ± 0.339	<0.001 *
WSSD (Pa/m)	13.715 ± 304.26	15.449 ± 250.67	-37.863 ± 622.06	<0.001 *
TransWSS (Pa)	0.034 ± 0.155	0.044 ± 0.140	0.111 ± 0.211	<0.001 *

* Abbreviations: WSS=wall shear stress, WSS_{Norm}=normalized wall shear stress, OSI=oscillatory shear index, RRT=relative residence time, WSSD=wall shear stress divergence, TransWSS=transverse wall shear stress, IQR=interquartile range.

Table S2: Detailed literature review of past studies exploring the relationship between hemodynamics and different IA wall types as observed intraoperatively

Author, Journal, Year	Sample Size	Image analysis	CFD Parameters	Findings (significant parameter[s])
Kadasi et al., J Neurosurg, 2013	n=16	Semi-automated	Inflow: Const. WSS<br>Outflow: N/A	Thin walls had low WSS, low WSS_{Norm} and high Pressure (WSS)
Sugiyama et al., Neurosurgery, 2013	n=30	Manual	Inflow: qMRA<br>Outflow: Zero pressure	Thick wall: Area with prolonged RRT co-localized with atherosclerotic wall. WSS low; not significant (RRT)
Suzuki et al., J Biomech Sci and Eng, 2015	n=4	Manual	Inflow: qMRA<br>Outflow: qMRA and ration of cross-sections	Thin wall: high WSS, thick wall: Low WSS and high RRT. (N/A)
Sugiyama et al., Comp Math Methods Med, 2016	n=8	Manual	Inflow: qMRA<br>Outflow: Murray's law	Thick wall: Atherosclerotic aneurysms had lower mean velocities and older blood. Lower WSS (N/A)
Suzuki et al., Neurosurgery, 2016	n=50	Semi-automated	Inflow: Generalized<br>Outflow: Zero pressure	Thin wall: High pressure difference is a key parameter for identifying thin-walled regions. (Pressure)
Talari et al., Asian J Neurosurg, 2016	n=9	Manual	Inflow: N/A<br>Outflow: N/A	Thin wall: Low WSS and high Pressure (N/A)
Cho et al., Biomed Res Int, 2018	n=21	Semi-automated	Inlet: Generalized<br>Outlet: Pressure Profile	Thin wall: Lower WSS. No association of OSI and pressure. (WSS)
Feletti et al., Acta Neurochir, 2018	n=18	Manual	Inflow: N/A<br>Outflow: N/A	Thin wall: low WSS and high pressure (N/A)

Furukawa et al., PLoS One, 2018	n=24	Manual	Inflow: Generalized Outflow: Traction free	Thick wall: Hyperplastic remodeling lesions had low WSS_{Norm}, AFI and high OSI and RRT (WSS_{Norm}, AFI, OSI, RRT)
Jiang et al., Neurosurg Rev, 2018	n=41	Semi-automated	Inflow: Generalized Outflow: Traction free	Thick wall: low WSS and higher low shear area. (WSS, WSS_{Norm} and LSA)
Cebral et al., AJNR, 2019	n=65	Manual	Inflow: Generalized Outflow: Murray's law	Thick wall: Low and oscillating WSS, Thin wall: high and less oscillating WSS (Pressure, WSS and OSI)
Jiang et al., J Clin Neurosci, 2019	n=28	Manual	Inflow: Generalized Outflow: Traction free	Thin wall: thin regions were associated with high pressure and low WSS. (Pressure, WSS_{Norm})
Kim et al., Neurosurgery, 2019	n=11	Manual	Inflow: Generalized Outflow: Traction free	Thin wall: High WSSD and high pressure could identify thin regions in aneurysms (N/A)
Kimura et al., World Neurosurg, 2019	n=12	Semi-automated	Inflow: Generalized Outflow: Traction free	Thin wall: Low WSS vector variations in a cardiac cycle can predict thin walled regions. (N/A)
Tanaka et al., J Neuroend Ther, 2019	n=50	Manual	Inflow: Generalized Outflow: Pressure zero	Thin wall: high WSSD regions coincided with thin regions. (N/A)
Ashkezari et al., JNIS, 2020	n=32	Manual	Inflow: Generalized Outflow: Murray's law	No difference in hemodynamic values between thin and thick blebs. (None)
Uchikawa et al., World Neurosurg, 2021	n=11	Semi-automated	Used PC-MRA for hemodynamics	Thin wall: Bleb redness was correlated with ratio of bleb to neck inflow velocities. (Velocity ratio)

* Median values of the median hemodynamic value at each wall type across all aneurysms. Abbreviations: WSS=wall shear stress, WSS_{Norm}=normalized wall shear stress, OSI=oscillatory shear index, RRT=relative residence time, WSSD=wall shear stress divergence, qMRA=quantitative magnetic resonance angiography, PC-MRA=phase-contrast magnetic resonance angiography, LSA=low shear area, AFI=aneurysm formation indicator.

Supplemental Figures

Figure S1

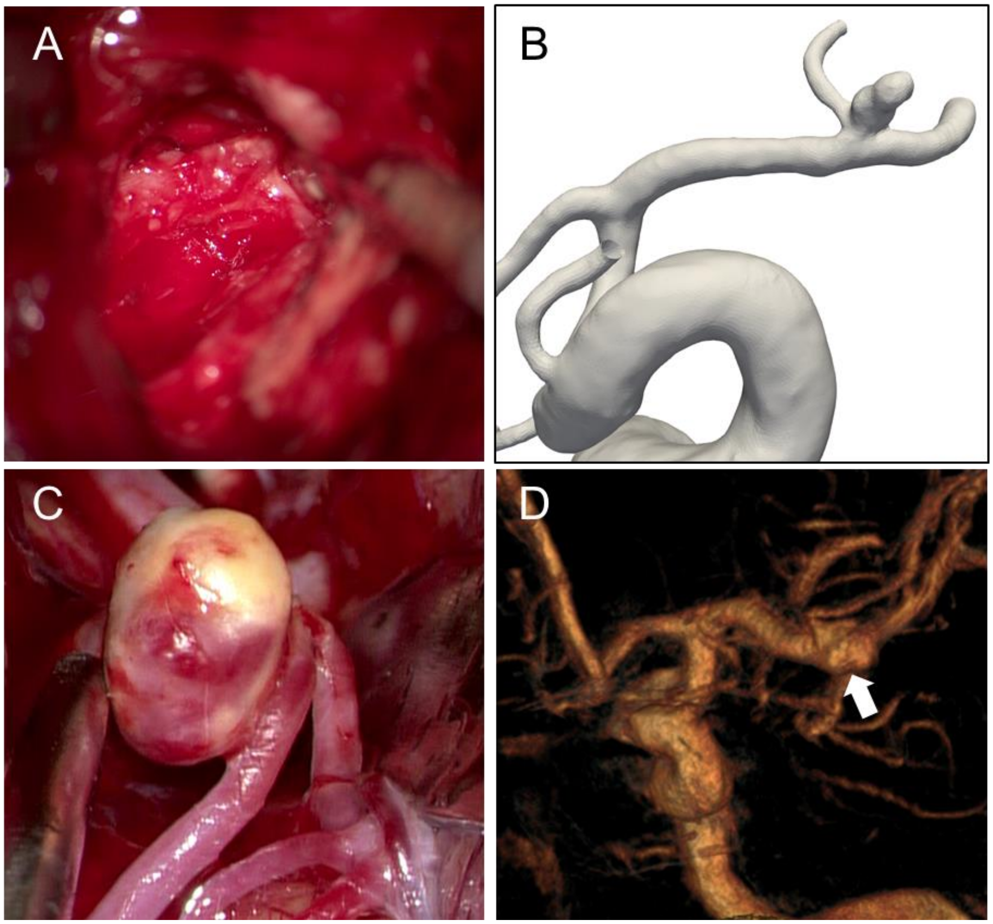

Figure S1: Examples of cases that were removed from analysis: In a few videos, although the corresponding DSA image was available, a clear view of the aneurysm along with the neck could not be identified due to the location of the IA **(A, B)**. Other cases were not analyzed if the image quality of the DSA was low and limited high-quality segmentation **(E, F)**.

Figure S2

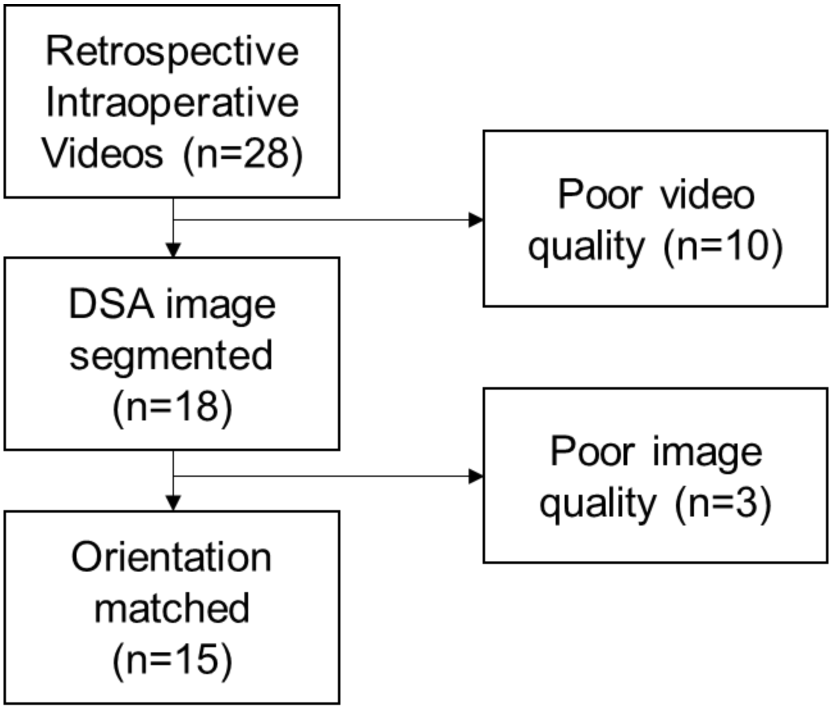

Figure S2: Data availability flow chart: In total 28 cases that had intraoperative videos between 2010 and 2016 were identified. Prior to analysis, we filtered out 10 cases because, while the IA was present, the majority of the aneurysm sac could not be seen on intraoperative video, and another 3 cases because after segmentation the quality of the geometry was not adequate for CFD. A total of 15 aneurysms were used for this study.

Figure S3

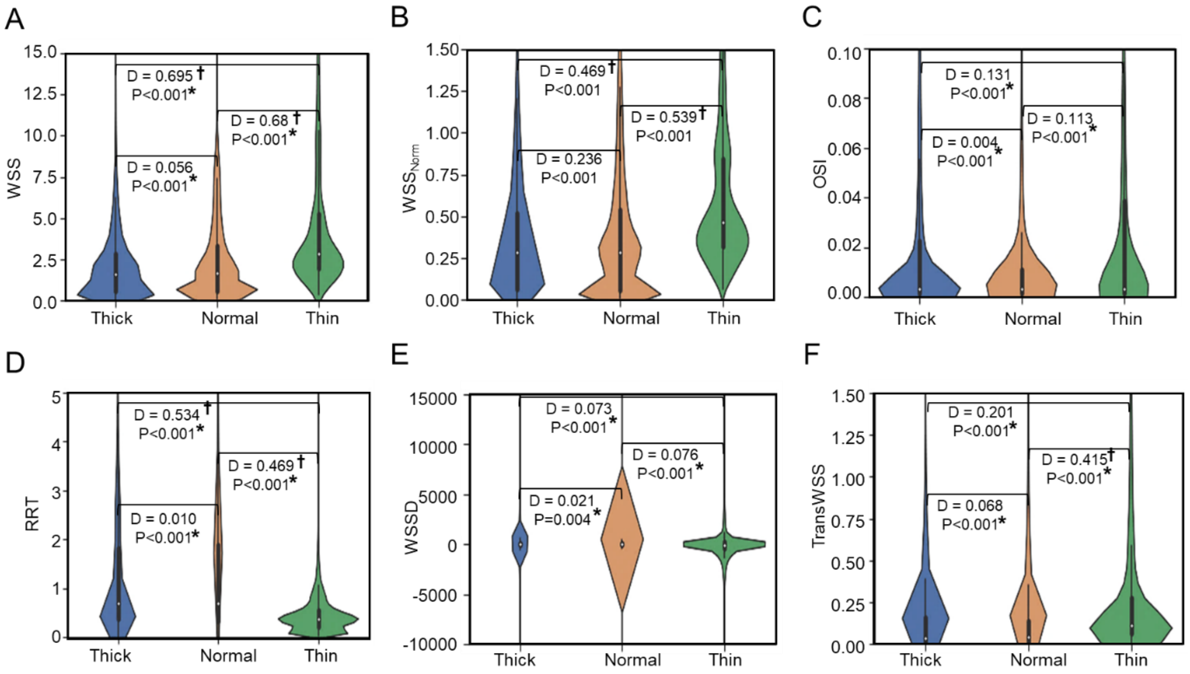

Figure S3: Pairwise univariate analysis of node-to-node hemodynamic variables between different wall types across all IA sacs: The violin plots of median values of WSS (**A**), WSSNorm (**B**), OSI (**C**), RRT (**D**), WSSD (**E**), and TransWSS (**F**) across all IA sacs. All the significant differences ($p<0.05$) are marked with an asterisk (*) and variables with a significant effect size ($D>0.4$) are marked with a **†.** Abbreviations: WSS=wall shear stress, WSSNorm=normalized wall shear stress, OSI=oscillatory shear index, RRT=relative residence time, WSSD=wall shear stress divergence, TransWSS=transverse wall shear stress.

Figure S4

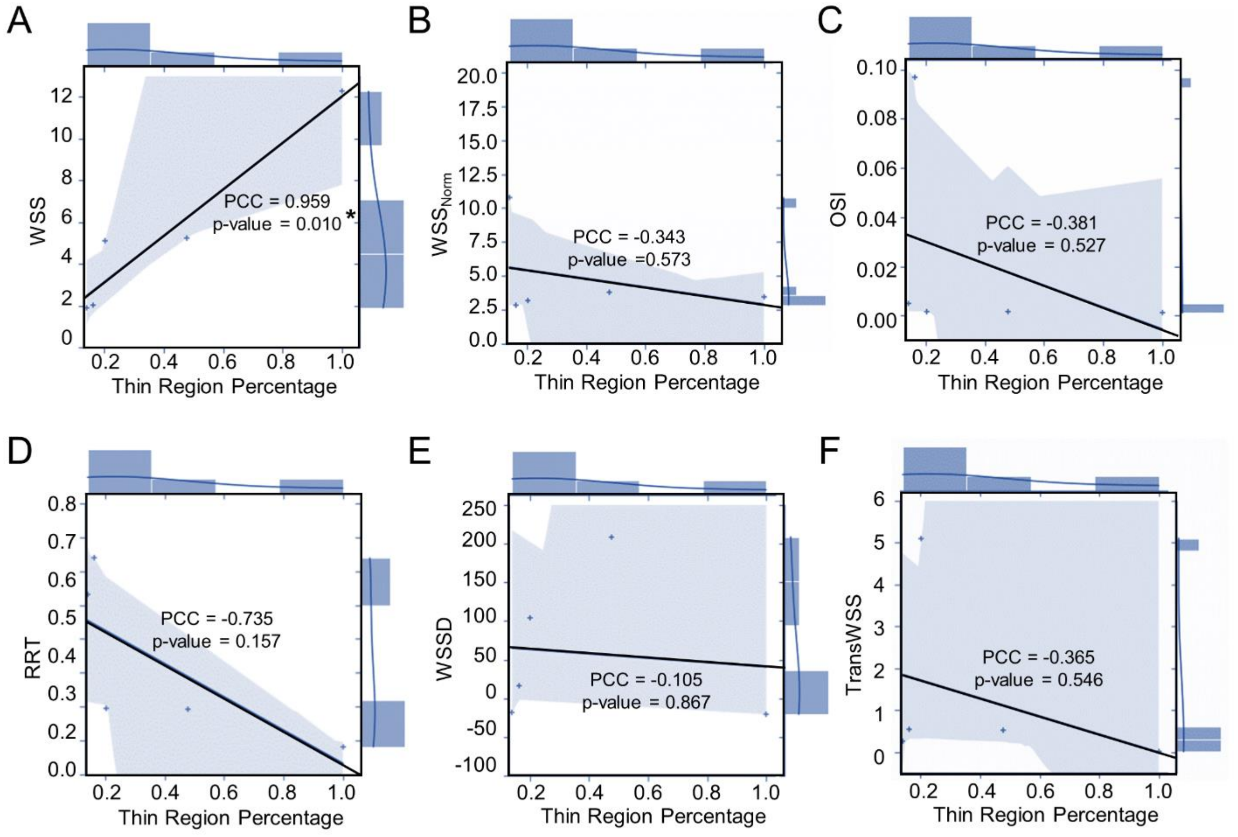

Figure S4: Regression analysis between hemodynamic variables and thin regions: The distribution of median values is shown via a histogram on the respective axes. The shaded regions represent the 95% confidence interval of the regression model bootstrapped over 1000 iterations. The PCC and the p-value from the Wald test is shown on each plot. The WSS had a significantly strong positive relationship with the percentage of thin regions on the IA sac. Abbreviations: PCC=Pearson correlation coefficient, WSS=wall shear stress, WSSNorm=normalized wall shear stress, OSI=oscillatory shear index, RRT=relative residence time, WSSD=wall shear stress divergence, TransWSS=transverse wall shear stress.

Figure S5

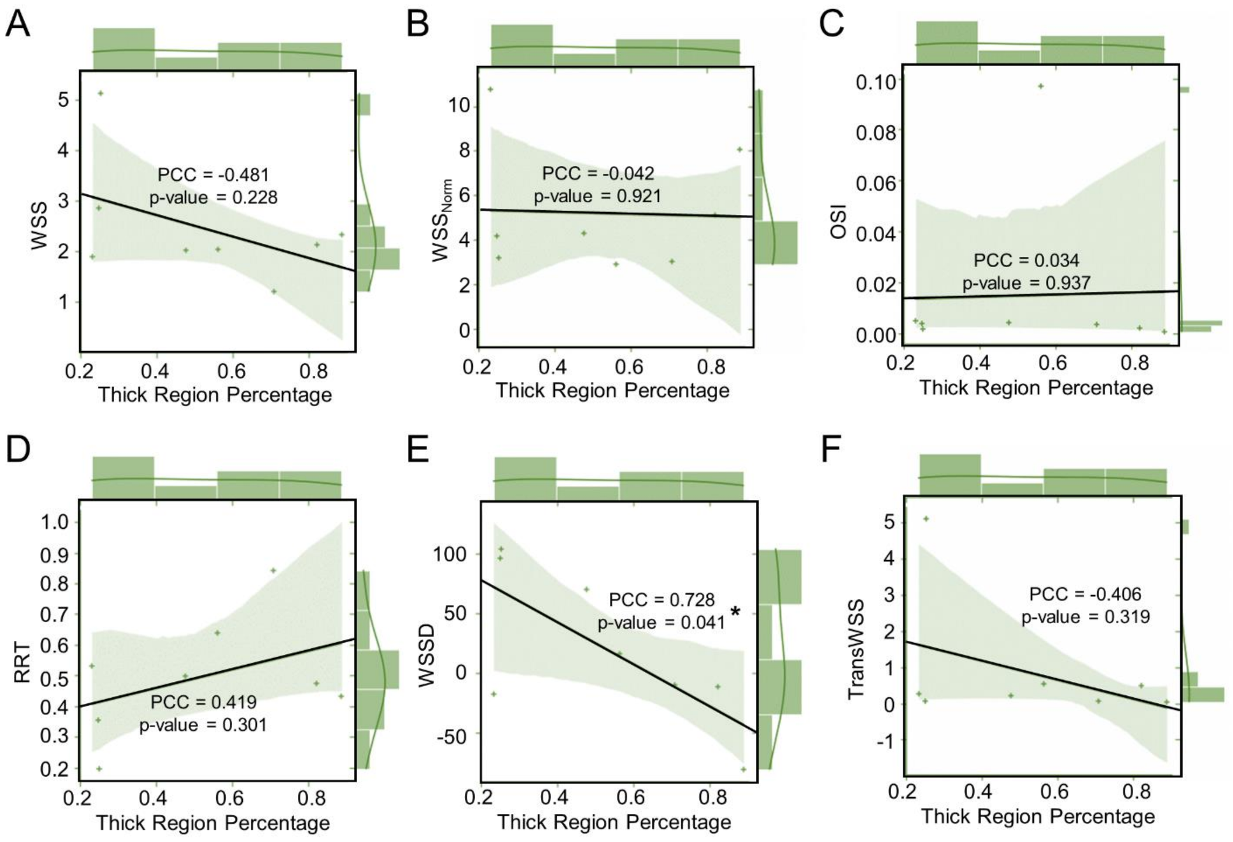

Figure S5: Regression analysis between hemodynamic variables and thick regions: The distribution of median values is shown via a histogram on the respective axes. The shaded regions represent the 95% confidence interval of the regression model bootstrapped over 1000 iterations. The PCC and the p-value from the Wald test is shown on each plot. The WSSD had a significantly strong negative relationship with the percentage of thick regions on the IA sac. PCC=Pearson correlation coefficient, WSS=wall shear stress, WSSNorm=normalized wall shear stress, OSI=oscillatory shear index, RRT=relative residence time, WSSD=wall shear stress divergence, TransWSS=transverse wall shear stress.
